# Virus-integrated laboratory models for habitability studies of Enceladus, Europa, and Titan

**DOI:** 10.1128/jvi.00904-26

**Published:** 2026-09-23

**Authors:** Damara Saggio, S. Chantal E. Stieber, Jamie C. Snyder

**Affiliations:** 1 Department of Biological Sciences, California State Polytechnic University - Pomona 6647, Pomona, California, USA; 2 Chemistry and Biochemistry Department, California State Polytechnic University - Pomona 6647, Pomona, California, USA; Indiana University Bloomington, Bloomington, Indiana, USA

**Keywords:** astrovirology, astrobiology, Europa, Enceladus, Titan, *Pyrobaculum*, Sulfolobales, Methanosarcinales, ANMEs, PFV, STIV, MetSV

## Abstract

Biogeochemical models have become critical for answering questions about the habitability of ocean worlds, especially that of early Earth. However, when extended to icy moons, experimental biogeochemical systems become especially challenging. Due to less constrained physicochemical parameters extrapolated from former flybys, the most comprehensive approaches could combine experimental and computational models of proposed ocean conditions with microbes present. This minireview makes the case for needing to also include viral/phage interactions with microbial models as a mechanism for maintaining biogeochemical cycling. The viral shunt, or the release and recycling of organic/inorganic matter in aquatic ocean systems via the viral lysis of bacteria and archaea, has proven to be a critical source of carbon/sulfur/iron cycling throughout Earth’s oceans. Nonetheless, the viral shunt remains understudied in the realm of other ocean world astrobiology and habitability studies but may provide a mechanism for detection of organics by future missions. The current knowledge of physicochemical conditions on three ocean worlds (Enceladus, Europa, and Titan) is briefly summarized in this work to contextualize examples of Earth-based integrated archaeal viruses (*Pyrobaculum* filamentous virus, *Sulfolobus* turreted icosahedral virus, and *Methanosarcina* spherical virus) that may be suitable for habitability studies for virus-integrated biogeochemical ocean world models.

## INTRODUCTION

Constrained by flyby data and computational studies, experimental geochemical models have become critical for establishing the potential habitability of ocean worlds, like Europa (1), Enceladus (2–4), and even early Earth/Mars (2, 5–8). However, there are few reports of geomicrobiological models with integrated chemolithotrophs (9–12), and even fewer models that include their viral pathogens. Given the role of the viral shunt in oceanic nutrient cycling on Earth (13, 14), integrating chemolithotrophs and their viral pathogens into other ocean world models provides the opportunity to probe nutrient cycling and habitability. Chemolithotrophs are particularly relevant to such models because they utilize inorganic redox substrates predicted for ocean worlds, thereby directly coupling microbial metabolism with surrounding geochemical cycles. More importantly, such models can generate testable life detection predictions, including those involving preserved nucleic acids, proteins, and lipids from viruses and their hosts. If the release of cellular and viral components mediated via the viral shunt occurs in ocean worlds, this mechanism may ultimately increase the likelihood of organic detection in future ocean world missions.

Archaea have long been posed as candidates for ocean world studies, given their mostly extremophilic physicochemical preferences and diverse metabolic capabilities (9, 15, 16). Among the most extensively studied archaea are the Sulfolobales, which are capable of metabolizing sulfides into sulfates in acidic hot springs and are potential microbial models for studying Europa’s once presumed H_2_SO_4_-rich ice shell (9, 16, 17). Viruses of the Sulfolobales are also some of the most studied lytic archaeal viruses and have ancient morphological characteristics (i.e., double jelly roll [DJR] capsid fold) that resemble lytic viruses that infect bacteria and Eukarya (18). Due to the diversity of viral genomics, probing viral markers shared among several domains via DJR can serve as a strong candidate in the search for astroviral signatures for life.

Viruses are the most abundant biological entities on Earth and are found wherever there is life, so this minireview argues that viruses should be included in habitability models of other worlds. Many archaeal viruses persist lysogenically (i.e., integrated within the host genome as a provirus), which may be advantageous under conditions of low host availability (19, 20). However, should environmental shifts stress the host cell enough (e.g., UV/temperature/pH shifts), temperate viruses that were previously dormant may become lytic, activating virion proliferation and eventual host cell lysis (20, 21). Lytic viruses are also critical in marine ecosystems, as their destruction of host cells can recycle over one-third of the ocean’s microbial biomass back into the ocean, i.e., the viral shunt (13, 14). Since Europa’s ocean is predicted to be twice the size of Earth’s (16, 22, 23), the viral shunt may play similarly critical roles in its ocean’s nutrient cycling and may contribute to unique biosignatures that could be detected in future missions, including viral particle detection.

This minireview begins by highlighting current efforts to probe the geochemical and physicochemical conditions of three ocean worlds: Enceladus, Europa, and Titan. Candidates for microbe-integrated ice-ocean models of said ocean worlds will then be presented with a viral pathogen capable of viral shunt-mediated nutrient cycling. This minireview guides the reader through design components for virus-integrated hydrothermal vent experiments by highlighting the chosen ocean worlds, reported ocean models, why specific bacteria and/or archaea should serve as the microbial models for their respective icy moons, why specific viruses should be included, and finally, how ocean world biogeochemistry can be further studied from an experimental biogeochemical perspective.

## WHY THESE TARGET BODIES?

The three target bodies, Enceladus, Europa, and Titan, are considered to be among the most promising ocean worlds for habitability in our solar system (24) and are therefore the focus of this minireview.

### Enceladus

The building blocks for life have been detected from active plume emissions excreting from Enceladus’ south-facing tiger stripes, making the Saturnian moon a primary target body for habitability research (21–23). In addition to what appears to be geothermal energy for life, the detection of H_2_ (25), C_x_H_Y_ (26), NH_3_ (27), and PO_4_^3−^ (28) by the Cassini-Huygens Ion Neutral Mass Spectrometer (INMS) established Enceladus’ subsurface ocean as a prime location for possible autotrophic and heterotrophic metabolism (Table 1). The detection of H_2_, SiO_2_, NH_3_, and CO_3_^2−^ in particular suggested potential for a reducing, alkaline ocean floor with serpentinizing hydrothermal rock-water chemistry, much like that of Earth’s Lost City Vents (25, 27, 29, 30).

**TABLE 1:** Summary of solutes detected and subsequent physicochemical dynamics proposed for Enceladus, Europa, and Titan^*c*^

Detected/proposed	Enceladus	Europa	Titan
H_2_^*a*^	X (25)	X (31, 32)	X (33, 34)
CO_2_	X (28, 29)	X (35–38)	X (39, 40)
CH_4_	X (26, 29)		X (41–44)
C_X_H_Y_	X (26)		X (45–48)
PO_4_^3-^	X (28)		
SiO_2_^*a*^	X (29)		
MgSO_4_		X (9, 49)	
H_2_O_2_		X (50)	
O_2_		X (37, 49–51)	
H_2_SO_4_		X (9, 36, 38)	
NH_3_/NH_4_^+^	X (27)		X (52)
Proposed pH^*b*^	Ocean: alkaline (25–29)	Ice shell lakes: acidic (9, 36, 37, 53) Ocean: alkaline (35, 36, 54)	HP slush: alkaline (46, 52, 55)
Temperature (°C)^*b*^	Ocean: −14 to 0 (56) Vents: warm (28)	Subsurface lakes: near 0 (53, 54, 57) Bulk ocean: −14-0 (35, 36, 54, 56, 58, 59) Vents: warm, 50–100 (17, 56)	HP slush: −23 to +27 (46, 52, 55)
Salinity^*b*^	~1%–3% (29, 60, 61)	~3%+ (49, 58, 62)	~1.0% (63)
Redox potential^*b*^	Ocean: reducing (25, 27)	Ice shell lakes: oxidizing (36, 50) Ocean: reducing (19)	Ice melt: reducing (46, 52)
Likely D.O. %^*b*^	Ocean: anoxic (22, 58)	Ice melt: microaerophilic (29, 36) Ocean: anoxic (41, 57, 64)	Ice melt: anoxic (52, 55)
Rock-water interactions^*b*^	Serpentinization (26–29)	Serpentinization (1, 45) FeS/FeOOH redox (38, 46, 54, 59)	Carbonaceous (45–48)
Metabolic possibilities^*b*^	Methane redox (26, 29) Fe redox (4, 65)	Methane redox (52) Fe/S redox (9, 29, 48)	Fermentation (46, 47) Methane redox (41–44)

^*a*^ Direct signs of hydrothermal activity.

^*b*^ Proposed, based on probe data and computational models.

^*c*^ Grey shading indicates a lack of presence in the target body.

Additional detection by Cassini’s INMS and Cosmic Dust Analyzer (CDA) revealed ions (Ca^2+^, PO_4_^3−^ Cl^−^, Na^+^, and Fe^2+^) indicative of apatite-containing rock water chemistry (26, 29), which has been argued as essential for early life formation (39, 40). While no sulfides have been detected so far on Enceladus, recent computational modeling supports the production of HS^−^ and subsequent FeS minerals (29). Given a liquid water-based ocean, hydrothermal energy for life, and the essential elements necessary for life as we know it (CHNOPS), Enceladus could be argued as the most habitable target body in our solar system. While NASA’s Orbilander probe containing a life detection suite (LDS) is still under development (66), microbially integrated ocean world models may be essential to predict biochemical signatures that could be detected in the future.

### Europa

Europa arose as a target body for habitability research when the Voyager probes imaged the presence of a young yet geologically active surface (67). Detailed images of cycloids, linnaea, and chaos terrain along the icy moon’s surface by Galileo further supported the potential for a thermodynamically active ocean below (58, 62). However, it was not until Galileo’s magnetometer revealed an induced magnetic field and a global density of 3.01 g/cm^3^ that a silicate core interacting with a global, salty, H_2_O(ℓ) based ocean below Europa’s icy shell was assumed (58). Near-infrared mapping (NIMS) and ultraviolet spectrometry instruments on Galileo further detected hydrated salts (H_2_SO_4_ and NaMgSO_4_) and oxidizing species (O_2_ and H_2_O_2_) on Europa’s surface, promoting the idea of a potentially oxidized and acidic ocean (9, 49, 50).

In response to Jupiter’s and Io’s gravitational field, tidal flexing within Europa’s mantle and ice shell are proposed, potentially promoting enough thermokinetic energy necessary to maintain subsurface ice shell lakes and a global subsurface ocean (68). However, recent computational studies of seafloor fluid flow showed insufficient fault activity for hydrothermal energy, consequently suggesting non-geothermal energy sources for life (51). Proposed subsurface lakes may be habitable zones as supported by surface oxidants and subsurface iron snow—iron-bearing mineral particles proposed to precipitate near the ice-ocean interface and sink into the underlying ocean—within Europa’s ice shell (17, 35). While Europa’s silicate seafloor is expected to be reducing, alkaline, and serpentinizing with 50℃–90℃ hydrothermal vent reactions (1, 22, 36, 69), the putative ice shell subsurface lakes are expected to be far more oxidized/acidic (37, 49, 50), near 0℃ (22, 35, 53, 56), and potentially sulfur rich (38). Provided O_2_ from surface radiolysis (37, 49, 50), H_2_SO_4_ from exogenous Io emissions of SO_2_ (36, 38), and iron snow from the ice-ocean interface (54), a unique habitable zone within Europa’s convective ice shell could potentially support aerotolerant acidophilic chemolithotrophs like Sulfolobales (70).

Ultimately, various proposed rock-water interactions (1, 57, 69), radiolysis-derived oxidative transport (37), and redox potential dynamics (54, 59) pose a wide range of conditions for Europa that still require extensive experimentation. Europa’s ice-ocean physicochemical conditions (Table 1) are not well constrained, resulting in a strong debate over how biochemical/geochemical laboratory models should be conducted (3, 60, 71). This minireview suggests using microbe- and mineral-integrated laboratory models to explore the possible physicochemical ranges/dynamics to better understand Europa’s habitability.

### Titan

Titan’s surface conditions, unveiled by the Cassini-Huygens probe, make this Saturnian moon a particularly unique world for potential habitability. Unlike Earth, Titan has frigid surface temperatures near −179°C (72), cryovolcanoes (64), methane clathrate lakes (41), and aerosolized tholins (42). Akin to Earth, Titan has a thick N_2_-rich atmosphere (72) and permafrost densely packed with CH_4_, NH_3_, and hydrocarbons (45). Hydrocarbons are proposed to leak from the methane clathrate lakes through cracks into the ocean as a nutrient source for fermenters (46, 47). NH_3_ detected by the Cassini-Huygens visual and IR mapping spectrometer proposed an alkaline subsurface ocean, with ammonia acting as an antifreeze agent (52).

Argon-40 detected via GC-MS in Titan’s atmosphere further supports geothermal activity and, by extension, the potential for hydrothermal activity in a subsurface ocean for Titan (48, 52). However, recent models of Titan’s alkaline subsurface revealed the likelihood of “slushie” high-pressure ice layers lined with small melt pockets, as opposed to a global subsurface ocean (55). Such systems would require microbes capable of withstanding wide variations in temperature (27 to −23℃), metabolites (amino acids, methane, carbon dioxide, and molecular hydrogen), and pressures in Ice III, V, and VI (Table 1; see Fig. 3). While still in development, Titan’s proposed robotic rotorcraft Dragonfly contains a Mass Spectrometer (DraMS) (46) whose data interpretation would benefit from laboratory experimental models with fermenters, methanotrophs, and methanogenic microbes in high-pressure, low-temperature systems, as is discussed herein.

## CURRENT ICY-OCEAN WORLD MODELS

Experimental ocean world models have been used sparingly in comparison to computational models to probe the origins and evolution of life on early Earth (2, 5–8, 10). Even fewer models have been applied to other ocean worlds to probe habitability (3, 69, 73). Early Earth models often are used to develop the potential hydrothermal and ice-ocean interface chemistries that could be occurring on said ocean worlds. For instance, Europa’s iron snow (54) and Earth’s iron-sulfur world hypotheses (74) both emphasize iron-rich minerals as a critical energy source for early chemolithotrophs (75). Consequently, iron-sulfur bearing hydrothermal chimney formation has long been a focus for understanding early Earth prebiotic chemistry (76). Experimental studies of early Earth alkaline vents with 5.5 pH, anoxic, and ferrous/ferric-rich solutions have involved injecting reduced sodium sulfide or sodium hydroxide effluent to probe the various minerals and redox potentials that could support life (5–8). Results have demonstrated that while iron hydroxide chimneys are more stable and thus easier to reproduce and analyze in a wet lab (7), iron sulfides promote more thermodynamically promising oxidation-reduction potentials (ORPs) for early Earth chemolithotrophy (5, 6, 77). This includes electrochemical analysis of diverse metabolic pathways, for instance, FeS oxidation (73, 78) and organic heterotrophy using ferredoxin enzymes densely packed with Fe-S clusters (74). The Jet Propulsion Laboratory’s success in growing early Earth hydrothermal vents and chimney models in a research lab (7, 8, 10, 79), combined with demonstrated microbe integration (10), influenced the efforts of recent Europa habitability models (17), though no further studies have been developed since then.

Hypotheses have been made regarding potentially habitable hydrothermal vent systems and ice shell subsurface lakes on Enceladus, Europa, and Titan. Plume emission spectrometry analysis indicated potential white smoker chemistries for Enceladus and Titan, composed predominantly of ammonia (Titan) (52), methane/organics (both) (41–44), dissolved silica (Enceladus) (29), and molecular hydrogen (both) (33, 34, 41, 42, 46). In contrast, Galileo’s gravity data (80) and NIMS data of Europa’s dark terrain (9) suggested possible FeS-based chemistry within Europa’s ice shell (60, 81). Each model system presents unique physicochemical limitations and is discussed in the following sections.

### White smoker models

**Fig 1:**
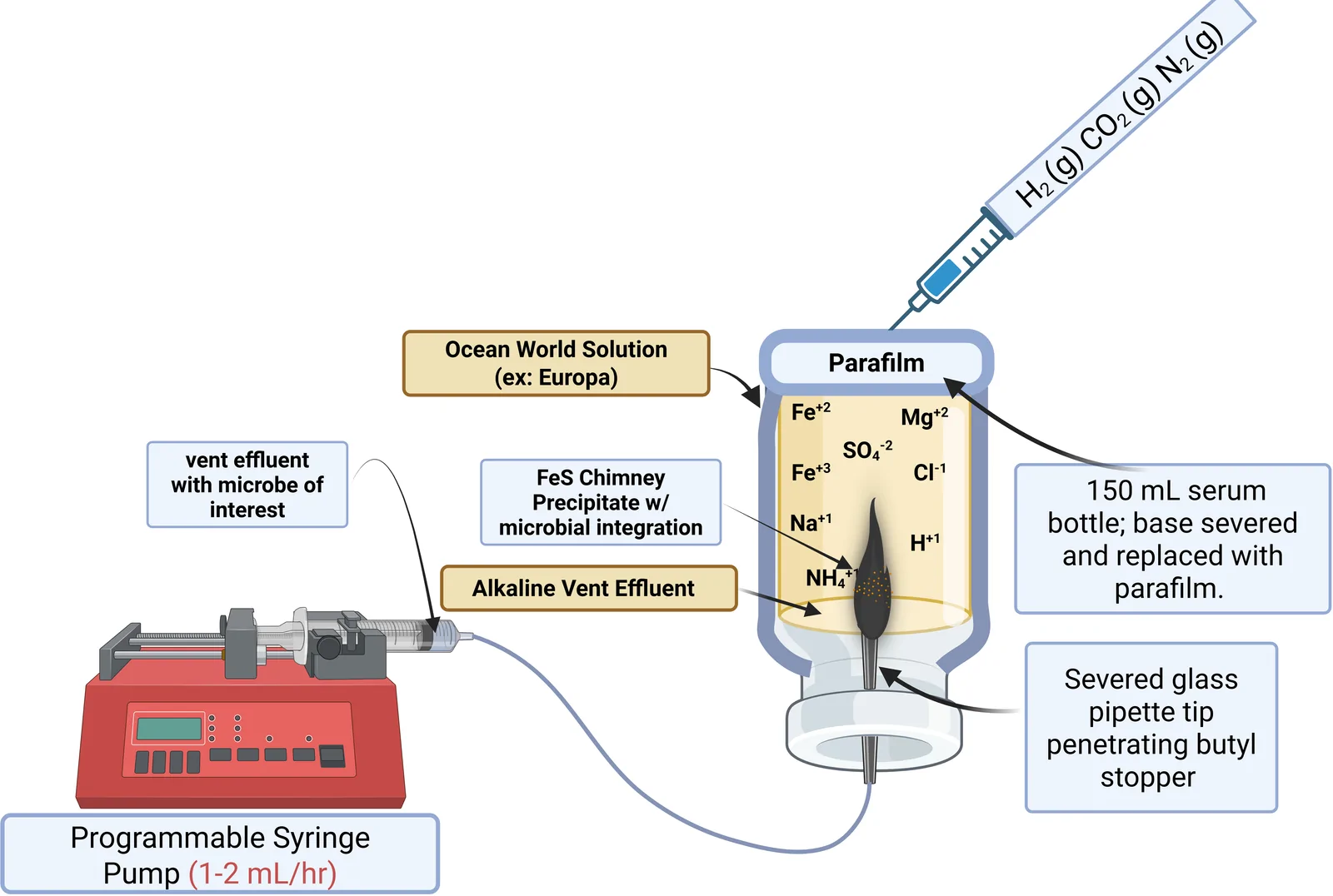
Diagram of historically applied laboratory vent model systems as described in previous studies (2, 6–8). The figure was created with BioRender.com and adapted from reference 17, previously published under a CC BY license.

Previous efforts to precipitate hydroxide and silicate-rich white smoker-like chimneys have involved alkaline effluent injections via a syringe pump into anoxic early Earth solutions (Fig. 1) (2, 6, 8, 82). Solutions mimicking that of early Earth’s ocean have included low pH (2–5.5) conditions at room temperature with 3.5% salinity and 10–60 mM concentrations of FeCl_3_·6H_2_O, FeCl_2_·4H_2_O, MgCl_2_·6H_2_O, and MgSO_4_, and in some cases, CaSO_4_·2H_2_O (2, 6, 8, 82). Alkaline vent effluent has included a range of 10–500 mM NaOH, Na_2_SiO_3,_ Na_4_SiO_4_, or silica-rich water glass set to pH 9–11 (2, 6, 8, 82). Model systems have ranged from ambient temperatures up to 70℃–90℃, which can be expected for white smoker effluent (6–9, 82). Solute concentrations used in these models often exceed those expected for early Earth, with the purpose of forming more robust chimney structures and faster precipitation (71, 76). Chimney formations have been analyzed via infrared (IR), Raman, scanning electron microscope (SEM), and powder X-ray diffraction (pXRD) and indicated the presence of magnesium silicates, iron phosphate silicates, iron hydroxides, and amorphous Fe/Mg materials in response to serpentinization leaching (2, 8, 82).

### Black smoker models

Previous efforts to precipitate FeS-based black smoker-like chimneys have involved injecting Na_2_S into ferrous-rich early Earth ocean-like solutions, resulting in the precipitation of pyrite, mackinawite, iron oxy-hydroxides, and greigite when the effluent was left at room temperature and the ocean system was heated to 75℃ (Fig. 1) (5–7, 82). Previous work on hydrothermal simulations also modeled precipitation predominantly under completely anoxic and less acidic (pH 5.5) early Earth conditions (5–7, 82). Vent effluent containing 10–50 mM sodium sulfide solution has predominantly been applied to simulate black smoker FeS-based precipitation, even though effluent is much more alkaline (pH 9–11) as opposed to acidic (5–7, 82). Thermal gradients have also been attempted with heating jackets, syringe heaters, and circulating chillers, but sustained vertical chimney growth with sulfide-bearing precipitate proved difficult to maintain compared to that of silicate/hydroxide-rich precipitate (6, 7). Mineral characterization with IR, Raman, SEM, and/or pXRD analyses has historically occurred under air-free conditions, with an emphasis on preventing environmental oxidation of the FeS during extraction and analyses (6, 7). While iron oxyhydroxides are commonly produced under room temperature hydrothermal vent conditions, we were unable to find reports that grew majority pyrite or greigite-bearing chimneys without using anoxic high-heat methods (6).

### Ice shell models

Europa’s surface ice habitability has been probed biologically with *Sulfolobus shibatae*, *Escherichia coli*, and *Deinococcus radiodurans* (9). Not surprisingly, results revealed that at low temperatures (80 K or −193°C) and pressures (10^−4^ torr), *D. radiodurans* had the highest survivability among the three microbes (9). However, the results remained inconclusive, given that excessive freeze-thawing may have been the cause of cell death in the strains. Moreover, while surface ice conditions were probed, cells were grown via chemoheterotrophy in nutrient-dense media (i.e., TGY for *D. radiodurans*, LB for *E. coli*, and sucrose/YE-Brock’s media for *S. shibatae*), although such complex macromolecules have not yet been discovered on Europa (9).

A recent study probing the habitability of subsurface lakes on Europa used NaCl-, FeSO_4_-, and MgSO_4_-rich subsurface lake fluid supplemented with 10% Brock’s media (with and without 0.1% sucrose) to assess the viability of a cold-adapted *Sulfolobus acidocaldarius* (*Saci*) lysogen and its viral pathogen, *Sulfolobus* turreted icosahedral virus (STIV) in such conditions (17). Temperatures between 1°C and 2°C were maintained within a glass-jacketed reaction vessel to mimic predicted subsurface lake temperatures, and iron sulfides were precipitated using saturated sulfides and iron snow vertically injected via a syringe pump (17).

The result of this study was the discerned viability of *Saci* via extensive biofilm in mildly acidic (pH 5) FeS-rich subsurface lake conditions, as well as downstream viability plating (17). Biosulfur globules and viral major capsid protein (MCP) were discerned as a potential biosignature for life (17). The viral shunt analysis via transcriptomics and proteomics revealed positive detection of viral mRNA (via RT-qPCR) and MCP in biofilm samples but not subsurface lake solvent aliquots (17). This study did not integrate stable isotope probing (SIP) of sulfur or carbon to probe the impact of viral shunt on biogeochemical cycling, nor did it test non-lysogenic *Saci* as a negative control for biogeochemical analyses over time with and without viral lysis. Nevertheless, these experimental studies demonstrated the potential for archaeal models in astrobiology.

## WHY MAKE ARCHAEA THE MICROBIAL MODELS?

**Fig 2:**
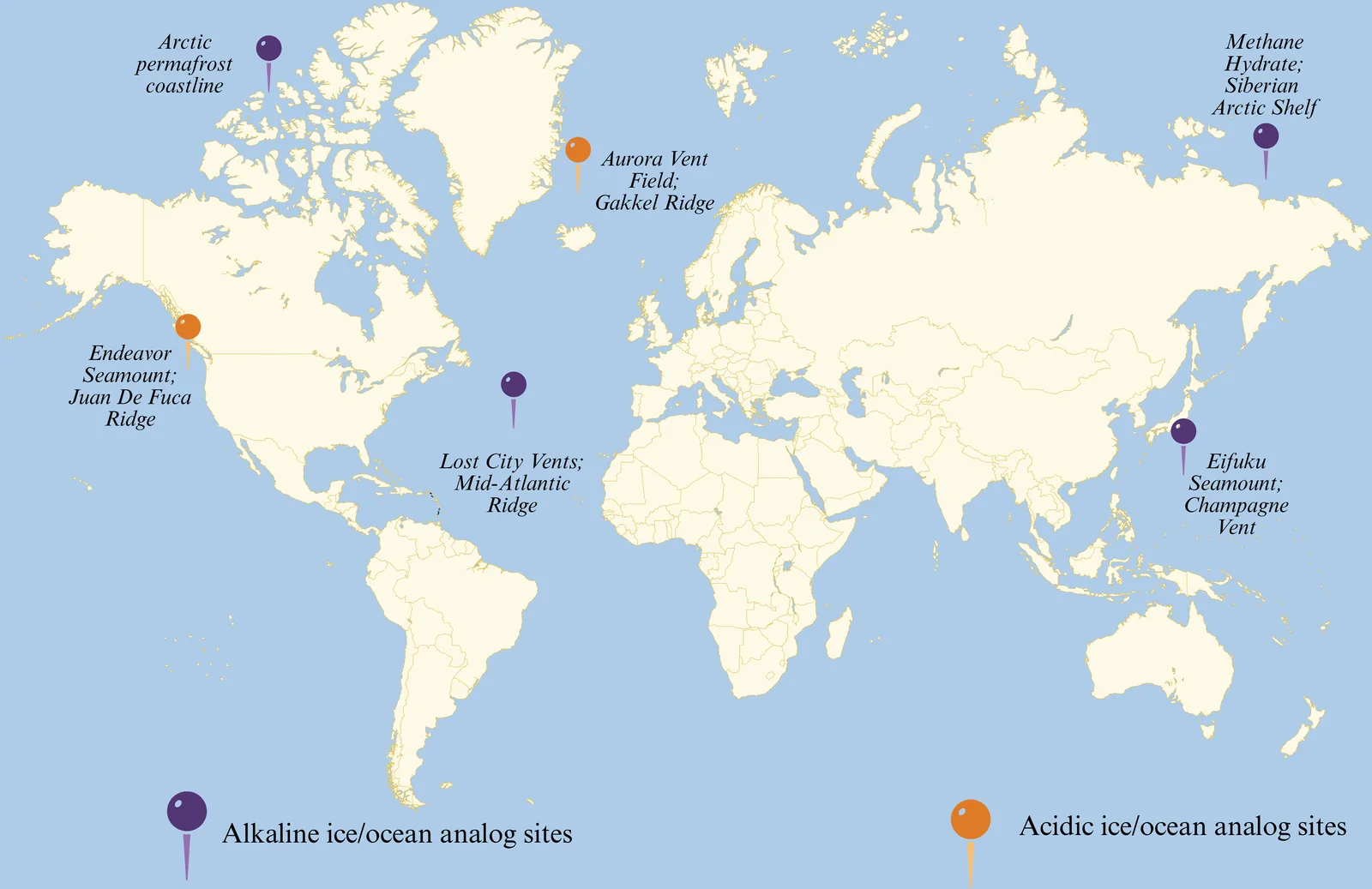
Map with example locations of Earth analog ocean vent ecosystems with extremophiles that thrive in alkaline (purple) and acidic (orange) conditions. The figure was created with BioRender.com.

While ubiquitous as mesophiles in soil systems and the human gut, archaea constitute a domain of life predominantly characterized by their extremophilic properties, having been isolated in extremely harsh environments around the world (Fig. 2) (74, 83). *Pyrobaculum* has been found in both hot springs and deep-sea marine vent systems in thermophilic, alkaline, and anaerobic conditions (84). Methane-producing Methanosarcinales can be found in hydrothermal marine systems and arctic permafrost rich in hydrocarbons (45, 85). Sulfolobales can be found in the hot springs of Yellowstone National Park (86), the volcanoes of Japan’s Owakudani (74), and the Solfataric fields of Italy’s Campi Flegrei (87). Many archaea, especially methanogens (44, 57, 69), have been posed as microbial models for astrobiology, given their ancient evolution, contribution to the rise of the eukaryote domain (83), and ability to survive via nutrient-limited conditions (86, 88–90). Many archaea have evolved not just to tolerate extremes but also to thrive and even depend on them, making their domain excellent for studying the “inhabitable” conditions predicted for other ocean worlds.

Archaea-virus models in this study were chosen based on their accessibility, their lysogenic properties, the extent to which their viral pathogen has been studied, and their physicochemical preferences in alignment with a target body of interest. Efforts were made to ensure that each microbe could be easily ordered on DSMZ/ATCC and that its lytic virus could be propagated for nutrient cycling/biosignature studies in target body conditions. While the majority of bacteria cultivated thus far express relatively mesophilic characteristics unlikely to survive the conditions being probed, the few extremophilic bacteria potentially capable of habitability—e.g., gamma/proteobacteria and sulfate-reducing bacteria (SRB)—have largely uncharacterized viral communities. As a result, model archaea with isolated and characterized viral strains are the focus of these habitability studies. Even still, syntrophic consortia, such as those found between methanogens, SRBs, and anaerobic methanotrophic archaea (ANMEs) in deep-sea vent systems, will be discussed for proposed Titan habitability studies. Consequently, this section will discuss how *Pyrobaculum*, Sulfolobales, and methane metabolizers—specifically their bacterial symbionts—could be possible microbial model systems for ocean world habitability studies of Enceladus, Europa, and Titan.

### Enceladus and *Pyrobaculum*

**Fig 3:**
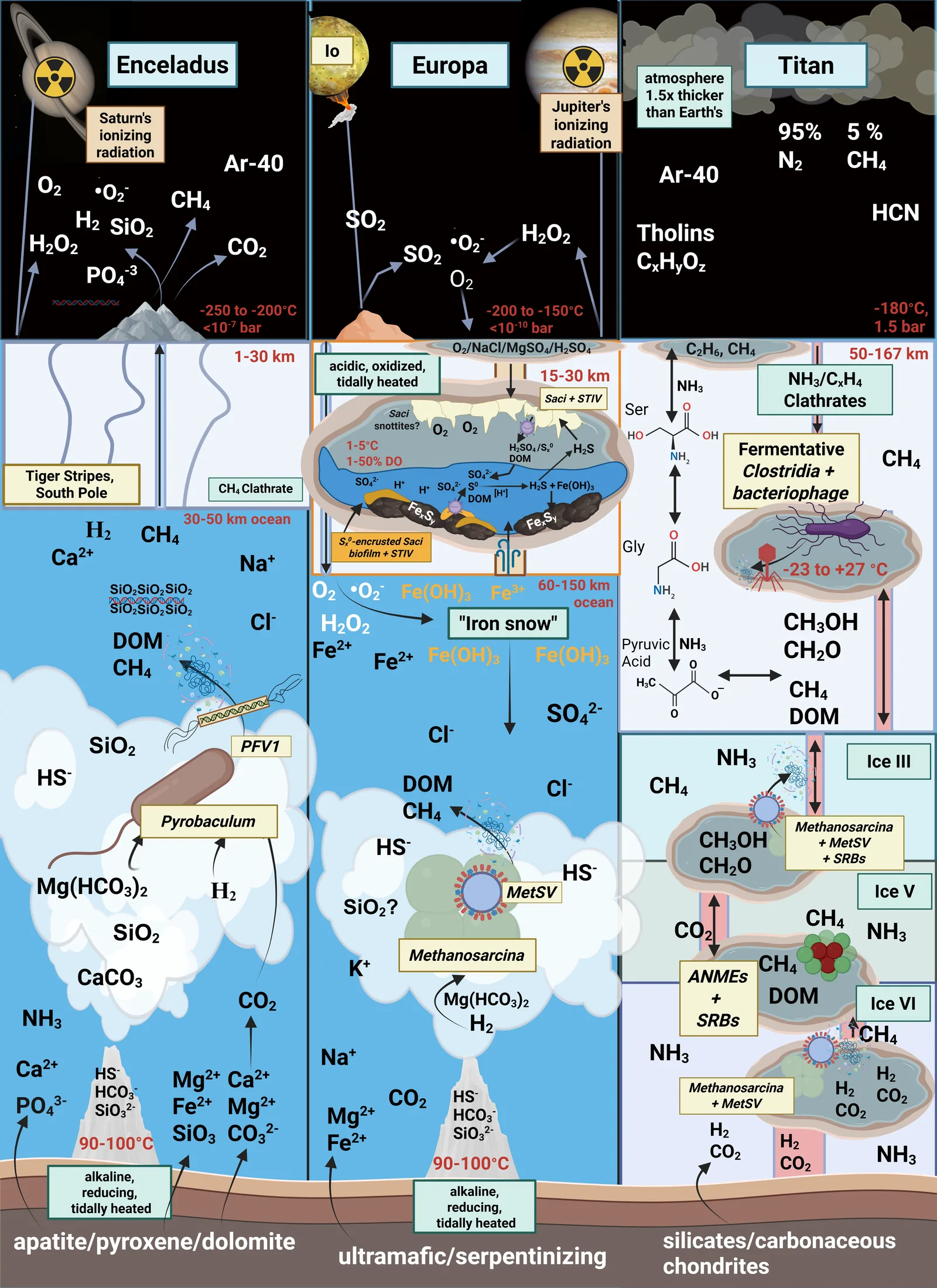
Proposed geomicrobiological models for Enceladus, Europa, and Titan. Archaeal (*Pyrobaculum*, *Sulfolobus*, Methanosarcinales, and ANMEs) and viral models (PFV, STIV, and MetSV) for each ocean world are shown alongside their potential biogeochemical cycles, with an emphasis on C/H/S sources and sinks from tidal heating, viral shunt, and hydrothermal emissions. The figure was created with BioRender.com.

With a silicate-rich mantle, Enceladus’ ocean is proposed to support serpentinizing rock-water interactions, resulting in a relatively alkaline pH, near 0℃ temperatures, supplemented with various solutes: PO_4_^3−^, SO_4_^2−^, H_2_, HCO_3_^−^, NH_3_, and CH_4_ (3, 25, 28, 60, 91). Cassini-Huygens INMS data (27) and Enceladus ocean modeling suggested CO_2_ emissions from the subsurface ocean and lower ocean salinities (1%–3%) compared to that of Earth’s 3.5% salinity (61). *Pyrobaculum* thrives in shallow marine hydrothermal vent ecosystems with alkaline pH and 1%–32% salinity; thus, the proposed white smoker vent systems on Enceladus could support *Pyrobaculum* metabolisms involving Fe, H_2_, HS^−^, and CO_2_ (84, 92, 93). *Pyrobaculum* is one of the most prominent archaeal populations surrounding shallow hydrothermal vents because it is capable of utilizing a variety of electron donors and acceptors, including H_2_, Fe(III), S_2_O_3_^2−^, and NO_3_^−^ (Fig. 3) (84, 92, 93). Isolation and propagation of *Pyrobaculum* under carbonated conditions using DSMZ Medium 611 also mimics serpentinizing ocean solutions with solutes like those detected from Enceladus’ plumes by Cassini-Huygens INMS, containing HCO_3_^−^, (NH_4_)_2_Fe(SO_4_)_2_·6H_2_O, and SiO_2_ at circumneutral pH (94). Under anaerobic conditions, *Pyrobaculum* can further be cultivated in Medium 611 using synthetic seawater mix, Wolin’s mineral solution, and trace ions all sparged with a gaseous mixture of 80% H_2_ and 20% CO_2_ (84, 94).

To probe the habitability of sulfur-reducing *Pyrobaculum* in Enceladus ocean conditions, DSMZ Medium 390—a well-defined culturing medium for anaerobic hyperthermophiles—can also be used (95). Provided with Allen’s trace minerals, thiosulfate, and sulfide in an anoxic solution, as a facultative anaerobe growing in media with up to 5% dissolved oxygen (D.O.) saturation, *Pyrobaculum* also provides opportunities to assess Enceladus’ potential biogeochemistry under microaerophilic conditions (84). While Enceladus’ D.O. saturations are not well constrained, predictions from ice surface radiolysis producing molecular H_2_ and O_2_ present intriguing possibilities for slight D.O. introduction, so models would benefit from a facultative anaerobe like *Pyrobaculum aerophilum* (60, 92, 93). Additionally, *Pyrobaculum* has a relatively short doubling time of 3 hours in optimal conditions, and a wide variety of strains are available via various cell culture banks, thereby facilitating *Pyrobaculum* as a model microbe for Enceladus studies for almost any research facility.

### Europa and Sulfolobales

A decades-long debate regarding the exogenous versus endogenous nature of sulfuric acid precipitates along its surface ice has left Europa’s ocean pH and subsequent redox potential unconstrained. While early studies proposed a globally oxidized (+mV), acidic subsurface ocean within a pH range of 2–6 (9, 37), recent studies have supported a more reducing and circumneutral ocean pH (54, 69, 96) with only the ice-ocean interface being slightly oxidizing and acidic due to brine vein exchange (54, 57; Fig. 3). Given its wide D.O. tolerance, acidophilic nature, minimal media requirements, and a long history of study in the realms of genetics, molecular biology, and biotechnology, Sulfolobales have become a model microbe beyond the focus of astrobiology (74, 97). Sulfolobales grow in a variety of minimal media, such as Brock’s media (70), ATCC Medium 1723 (98), and DSMZ Medium 88(a) (99). While Europa’s magnesium, iron, and sulfate ocean solute concentrations are not well constrained (60), early Earth’s metal-rich ocean conditions (7), and current black smoker effluent concentrations at Gakkel Ridge (100) and Juan De Fuca Ridge (101) (see Fig. 2) provide plausible starting concentrations for hydrothermal vent models on Europa.

However, recent computational modeling of Europa’s subsurface systems revealed limited tidal flexing within the mantle, thereby reducing the likelihood of hydrothermal energy for life on the seafloor (51). Consequently, subsurface lakes within the ice shell, predicted to be H_2_SO_4_ rich (pH 2–5.5) from exogenous Io emissions (38), oxidizing (1.5%–24% D.O.) from ice radiolysis (37, 49), and Fe/Mg/S rich from saturated brine veins meeting at melt pockets (102), also possess potential for Sulfolobales as a model microbe for studying Europa’s habitability. The doubling time of Sulfolobales depends highly on the reducing and oxidizing agents available (88). In heterotrophic conditions, the oxidation of tryptone, sucrose, or yeast extract often promotes relatively fast growth of approximately 4–8 hours per generation (103). Meanwhile, in autotrophic conditions, reducing agents such as elemental sulfur, hydrogen sulfide, and pyrite may require 24 hours to several weeks until the log phase of Sulfolobales is reached (78, 88). D.O. saturation also impacts the generation time for Sulfolobales (88, 104). Since the optimal D.O. is between 1.5% and 24% for some Sulfolobales (104), assigned D.O. saturations for Europa’s ocean will impact both pyrite mineralization and *Sulfolobus* proliferation (102).

Galileo’s near-infrared images of Europa’s dark terrain mirrored reflectance patterns of hydrated magnesium sulfate and sulfuric acid seen in Brock’s media, making it easier to grow *Sulfolobus* in Europa’s upper ice shell conditions (9, 49, 70). While Sulfolobales grow optimally at 75°C, they can withstand a wide range of 55°C–0°C and have even demonstrated prolonged dormancy at 0℃ (105), enabling a range of experimental conditions to be probed for habitability studies. A recent study also showed that when selected for and passaged over several months at near 0°C, *Saci* can become cold adapted over time; however, studies are still being done to understand the proteomic and genetic underpinnings of its cold adaptation methods (17).

Sulfolobales normally persist in freshwater hot spring environments with higher D.O. levels than those of deep-sea vent environments, but they have also demonstrated microaerophilic tolerance of 1.5%–24% D.O. (104) and halotolerance (400 mM NaCl) in recent studies (103). The same recent study also demonstrated *Saci* biofilm growth at 600 mM NaCl and 9.5%–31% final D.O., supporting higher halotolerance than initially conceived for these freshwater microbes (17, 73). Predictions surrounding microbial biofilm and its ability to act as an osmo-regulator for halotolerance (106), thermal insulator for psychrotolerance (107, 108), and nutrient reservoir have yet to be determined. However, interesting possibilities regarding *Saci*’s ever-evolving limits of life have been recently reported (17, 89, 103, 105).

In addition to using extracellular polymeric substances (EPS) as a thermal insulator and osmo-regulator (73, 106, 107), biofilm has been shown to act as a physical and chemical barrier to ionizing radiation (109, 110). This is especially pertinent to subsurface ejections on Europa’s surface, which would be bombarded by Jupiter’s magnetic field (49, 50). The thicker the EPS matrix, the more UV and other ionizing radiation can be scattered as opposed to penetrating cellular membranes and damaging DNA (109–111). EPS is also known to contain antioxidants that can act as radical scavengers, which is highly necessary for survival on the surface of Europa. This includes biosulfur globules whose polysulfides (S_n_^2−^) can scavenge radical oxidizing species for anaerobic microbes in oxidizing conditions (112).

Europa Clipper’s surface dust analyzer (SUDA), containing a time-of-flight secondary ion mass spectrometer (TOF-MS), is the most likely payload instrument to detect potential EPS or sulfur globules ejected in ice grains onto the surface, come 2031 (16, 23). With a mass range of up to approximately 10–10,000 Da, biosulfur ions alongside small sulfur-organics could potentially be detected by SUDA (16, 23). Experimental ice-ocean models that employ LD/TOF-MS to detect biogenic metabolites can therefore be useful for unpacking future Clipper data of ice grain bombardment.

### Titan and Methanosarcinales/ANMEs

While early models of Titan predicted a global water ocean (113, 114), recent studies include various high-pressure ice layers containing gas seeps, slush regions, and melt pockets throughout (55). While no O_2_ was detected by Cassini-Huygens, ammonia (NH_3_), H_2_O, N_2_, hydrogen cyanide, S, K, Na, and radiation-induced hydrocarbon polymers (tholins) were detected with linear and aromatic hydrocarbons in Titan’s atmosphere (41, 42, 64).

Hydrolysis of tholins in the convective surface ice can produce high yields of glycine (46, 47), an essential amino acid (Fig. 3). Via biogenic fermentation, tholins and their amino acid derivatives (e.g., Gly and Ser) could be metabolized by a variety of bacteria and archaea, including fermentative bacteria like *Clostridia* (46, 47, 115, 116). Ubiquitous in nature, *Clostridia* can be found in the human microbiome, anoxic soil, and even deep-sea vents (115, 117). *Clostridia* are also a good option as a Titan-specific fermenter because they can withstand relatively toxic levels of ammonia (5%–35%) in anaerobic culture (118). Titan’s slush is proposed to be alkaline due to predicted concentrations of dissolved ammonia (27, 52). Even though the salinity is predicted to be <1-3% (63), Titan’s subsurface water ice is expected to be anywhere from 0℃ (63) to temperate (max 27°C) depending on ammonia concentrations (55). While not an archaea, certain strains of *Clostridia* have demonstrated broad tolerance ranges relative to pH (4.6–10.2) (119, 120), temperature (3°C–42°C) (121), and salinity (1%–2%) (122), making it a strong candidate for Titan habitability research.

Subsurface NH_3_ is suspected to have accumulated during Titan’s initial formation (123), resulting in surface decomposition and a nitrogen-rich atmosphere (63, 124). As the NH_3_ ice gradient rises with higher pressures, so does the concentration of geogenic H_2_ and CO_2_ for methanogenesis (63). In high-pressure (V/VI) ice layers, outgassing from carbonaceous chondrites into melt pockets may be the source of chemical energy (63, 123). *Methanosarcina* are psychrophilic methanogens that have been isolated in arctic marine environments (71, 125) and hydrocarbon-rich permafrost (45). Methanosarcinales can also withstand Titan’s proposed NH_3_- and CH_4_-rich ocean via methylamine, methanol, and methyl sulfide metabolism (125–127).

Provided with H_2_ and CO_2_, *Methanosarcina* could fuel methane seeps traveling to the ice layers above, possibly even feeding methane oxidizers like ANMEs (Fig. 3) (85). ANMEs have been found deep within submarine permafrost, producing methanol and formaldehyde from methane trapped in the ice (85, 128, 129). SIP has revealed ANME activity via ^13^C-labeled saturated methane seeps (128, 130). Using methanol as an alternative carbon source for methanogenesis, *Methanosarcina* may also survive in the lower-pressure ice layers (III), interacting with metabolites from both fermenters and ANMEs (Fig. 3). On Earth, SRBs commonly form syntrophic consortia with ANMEs and/or methanogens, coupling methane metabolism to sulfate reduction (131). Consequently, incorporating SRBs into Titan analog laboratory models could further link methane and sulfur cycling and expand the range of potential biosignature predictions before the Dragonfly mission begins its data collection.

By modifying DSMZ’s *Methanosarcina barkeri* media (120a) (132), which contains methanol and/or bicarbonate degassed with a CO_2_/H_2_ source set to circumneutral pH, one may begin to develop an anaerobic Titan ice-ocean melt pocket model system (125). The doubling time of Methanosarcinales varies widely from 6 to 30+ hours, depending on the source and availability of carbon (126). In the presence of H_2_/CO_2_, *M. barkeri* can double in just 6 hours (11), while in the presence of methylamines, *M. barkeri* growth can take up to 30 hours (133). Should *Methanosarcina* be inoculated planktonically into Titan ice-ocean vent models, a range of carbon sources and pressures should be probed at 1°C–20°C to assess for microbial viability (41, 42, 63).

Dragonfly’s DraMS has GC-MS and laser desorption capabilities to detect both volatile and biologically relevant compounds (134). Using GC-MS, ^13^C-labeled saturated methane seeps may be detected, indicating the possibility of ANME activity (124, 128, 130). Meanwhile, methane-producing *Methanosarcina* can lead to ^12^CH₄-enriched methane, thus creating ^13^C/^12^C ratios in methane that could indicate active or past microbial methane cycling (128, 130).

## VIRUS-INTEGRATED OCEAN WORLD HABITABILITY MODELS

In Earth’s oceans, the biogeochemical cycling of CHNOPS elements is significantly impacted by virus-mediated lysis, which release of dissolved organic matter (DOM) and inorganic matter from bacteria and archaea (13, 14, 96). Recent studies have revealed that archaeal viral lysis accounts for around 0.5 gigatons of marine DOM that is recycled each year, approximately equivalent to one-third of the total recycled microbial biomass (14). Consequently, detecting the presence of viral particles of archaeal models will prove critical for probing the biogeochemical cycling and nutrient availability possible on other ocean worlds.

### Enceladus and *Pyrobaculum* filamentous virus (PFV)

A virus that could match a *Pyrobaculum* model for Enceladus studies is the *Pyrobaculum* filamentous virus (PFV) previously isolated in shallow marine vent systems and sulfurous fields surrounding volcanoes near Pozzuoli, Italy (87, 135). Within its hosts, such as *Pyrobaculum arsenaticum 2GA* and *Pyrobaculum oguniense TE7* (DSM13380), only lytic replication cycles of PFV have been recorded, indicating that the virus does not integrate into the host’s genome (87, 135). Negative controls are possible from several known *Pyrobaculum* that are not permissible to PFV (e.g., *Pyrobaculum calidifontis* VA1 [DSM 21063] as compared to those that are, such as *Pyrobaculum arsenaticum* PZ6 [DSM 13514]) (87). This provides suitable archaeal-virus systems where one system is capable of the viral shunt and nutrient cycling, and the other system is unable to cycle nutrients via the viral shunt.

PFV is a strong model for ocean world studies in that its enrichment cultures, transformation, and extraction techniques for sequencing have been defined (87, 136). These established procedures enable the feasibility of using *Pyrobaculum* for novel research areas, such as habitability research. What is currently unknown about PFV is its method of assembly, lysogenic capabilities, and whether or not it can remain intact extracellularly for extended periods (87, 136). Using environmental ocean world ranges for temperature, pH, and salinity thus has the potential to impact both habitability studies and our understanding of growth-limiting conditions for PFV. Ultimately, efforts to probe these aspects of PFV may aid in developing stronger virus-integrated ocean world experiments with potential applications to Orbilander probe experimental design.

Enceladus plume emissions have revealed silica (29), a compound commonly used in DNA extraction columns to bind and wash DNA prior to sequencing. Subsequent mechanisms of DNA/RNA preservation via silica encasement have been proposed (137, 138), some with astrobiological implications (139). Thus, models of Enceladus undergoing viral shunt should emphasize methods of DNA/RNA extraction with silica to determine how genetic biosignatures could be targeted instrumentally via Orbilander.

Provided with an external archaeal lipid envelope during viral budding (i.e., exocytosis-like exiting of the cell), PFV may also be flash-freeze tolerant following methods of plume-ejection studies (55). Monolipid ether-linked archaeal lipids, or glycerol dialkyl glycerol tetraethers (GDGTs), have evolved to extreme conditions to maintain their rigidity in high-temperature conditions, as well as their flexibility in low-temperature conditions (140, 141). Psychrophiles, for instance, have adapted isoprenoids with lower volumes of cyclopentane and cyclohexane rings for less dense, more porous membranes at low-temperature conditions (140, 142, 143). Meanwhile, thermophiles that require rigidity have adapted unsaturated, cyclopentane-containing pores, and GDGTs to increase packing and reduce porosity for stronger membranes (143). Provided the viral shunt, reduced sedimentation of microbial DOM on Enceladus may promote more lipid biogeochemical cycling, ultimately increasing the chances of biosignature detection during future flybys of the Saturnian system.

### Europa and *Sulfolobus* turreted icosahedral virus (STIV)

The STIV family of viruses is selected as a potential virus in Europa models because they are found extracellularly in oxidized, sulfuric acid-rich conditions similar to that of Europa’s proposed subsurface lakes (17). STIV has one of the most acid- and heat-resistant lipid envelopes underneath its MCP, ultimately promoting structural and genomic stability in extreme conditions, like that of Europa’s ice shell surface (20). STIV also is of interest because certain variants (STIV2 and STIV3) can integrate and then excise from the host cell genome (via lysogeny) in the event of host cell stress, such as sharp changes in environmental UV, pH, temperature, or nutrient exposure (20, 144–146). These stressors may be at play on Europa, so such virus-host interactions make STIV/*Sulfolobus* a unique microbial pair for Europa ocean habitability studies.

With dense and symmetrical MCP-containing morphologies, partial instead of whole STIV damage is more likely, resulting in the possibility of plume ejection survival. While Europa Clipper may not have the mass spectrometry (MS) capabilities to measure 37 kDa-sized MCP particles, its SUDA instrument may be able to detect fragments of an MCP, such as sheared portions of the eight beta-barrel bands. Virions containing an internal lipid membrane, as seen in STIV, can further protect its dsDNA from ionizing radiation. The Europa Clipper’s SUDA, containing a detection range of ~10–10,000 *m*/*z*, has the capacity to detect said lipid and DNA contents (16, 147). Given that STIV’s lipid membrane is derived from its thermoacidophilic archaeal host, archaeal virus analogs may prove especially durable to large pressure changes upon surface ice ejection studies. While the amount of cyclopentane rings may vary within archaeal lipid GDGTs (e.g., GDGT-0 has no cyclopentane rings, while GDGT-4 has four), SUDA may very well be able to detect lipids with or without said rings. Specifically, biphytanyl chains with and without rings may be distinguished with a unique MS fingerprint.

### Titan and *Methanosarcina* spherical virus (MetSV)

For a model of Titan’s subsurface aqueous ocean, *Methanosarcina* spherical virus (MetSV) is a possible option, as it has been isolated from a variety of anaerobic environments, including anaerobic sewage digesters, alkaline mud from drilling sites, and biomethanation granules (148). It has a relatively fast (4-hour) replication cycle preceding cell lysis (148), which is beneficial for experimental expediency. *Methanosarcina mazei* strains are also easily accessible through DSM culture banks within DSMZ Medium 324 for anaerobic cultivation under CO_2_/N_2_ without agitation (148).

One challenge with MetSV is that it is not widely studied, and it may take several cycles to concentrate MetSV sufficiently for laboratory modeling of Titan (148). MetSV’s methods of replication and assembly, as well as other potentially susceptible methanotrophic hosts, are also another unknown for this archaeal virus. Therefore, the choice of MetSV in Titan habitability studies can benefit both viral understanding and habitability frameworks of other worlds. Similar to STIV, MetSV also has an internal lipid envelope, increasing the durability of a viral genome at the lower-level/high-pressure ice systems. Dragonfly’s DraMS has a mass detection range of ~50 to 2,000 Da. Consequently, intact GDGTs (~1,300 to 1,500 Da) dissolved within the methane lakes could theoretically be detected by DraMS; nonetheless, intact MCP may prove more difficult (134). Provided with repeating peptide mass patterns consistent within an MCP, high frequencies of specific amino acids matching an analog MCP composition (from MetSV’s genome or proteome) could perhaps be coupled with the detection of internal lipid fragments, ultimately hinting at a virion within a Titan subsurface-slush experimental model.

## HOW CAN NUTRIENT CYCLING BE STUDIED?

The aforementioned archaeal-viral systems enable nutrient cycling to be studied, with a common approach being SIP using ^13^C- and ^34^S-labeled nutrients. Previous studies of virus nutrient cycling demonstrated that cycling can be tracked to better understand the dynamics of matter and energy in each ocean system (14). As shown in Fig. 3, the viral shunt could strongly influence geochemical cycles in ocean systems described in this minireview, ultimately recycling DOM back into the ocean and potentially out via plume emissions. By comparing systems with and without the viral shunt via SIP, we can track any differences in potential biosignatures and discern what types of astrovirological biomarkers could be probed in the search for life beyond Earth.

By 2031, the Europa Clipper space probe is expected to conduct flybys through Europa’s thin atmosphere and potential plume emissions (16, 23). IR and MS data will be recorded with the overall objective of further unveiling the icy moon’s physicochemical conditions (pH, ORP, and temperature) and probing its habitability for life as we know it (16). Thus, in anticipation of Clipper’s Mapping Imaging Spectrometer for Europa and Mass Spectrometer for Planetary Exploration payload instruments, IR and MS analyses should be used in microbe-ocean world models to assess what signatures of rock-water chemistry, chemolithotrophic metabolites, and microbial fragments Clipper might detect before it reaches the Jovian system (16). This includes using MS to probe stable isotope dynamics with and without viral models using ^34^S, ^13^C, and ^57^Fe to study the effects of viral shunt on biogeochemical signatures on each icy ocean moon.

## CONCLUSION

This minireview highlights the ice-ocean conditions of Enceladus, Europa, and Titan and pairs them with archaea and virus-integrated ice-ocean world models that could be used to create experimental systems that maintain biogeochemical nutrient cycling. A few examples of extremophilic archaea and viruses were discussed here to represent possible analog systems that could be applied in habitability studies of other worlds due to their ability to thrive under extreme Earth conditions. It is expected that continuing archaeal/viral integrated icy ocean model research on Earth will present new archaeal/viral pairs, where the inclusion of a virus is critical for nutrient cycling and DOM availability for long-term habitability.

## References

[1] Thompson SP, Kennedy H, Butler BM, Day SJ, Safi E, Evans A. 2021. Laboratory exploration of mineral precipitates from Europa’s subsurface ocean. J Appl Crystallogr 54:1455–1479. doi:10.1107/S1600576721008554 PMC8493616 34667451

[2] Barge LM, Doloboff IJ, White LM, Stucky GD, Russell MJ, Kanik I. 2012. Characterization of iron-phosphate-silicate chemical garden structures. Langmuir 28:3714–3721. doi:10.1021/la203727g 22035594

[3] Barge LM, White LM. 2017. Experimentally testing hydrothermal vent origin of life on Enceladus and other icy/ocean worlds. Astrobiology 17:820–833. doi:10.1089/ast.2016.1633 28836818

[4] Roche MJ, Fox-Powell MG, Hamp RE, Byrne JM. 2023. Iron reduction as a viable metabolic pathway in Enceladus’ ocean. Int J Astrobiology 22:539–558. doi:10.1017/S1473550423000125

[5] Mielke RE, Robinson KJ, White LM, McGlynn SE, McEachern K, Bhartia R, Kanik I, Russell MJ. 2011. Iron-sulfide-bearing chimneys as potential catalytic energy traps at life’s emergence. Astrobiology 11:933–950. doi:10.1089/ast.2011.0667 22111762

[6] White LM, Bhartia R, Stucky GD, Kanik I, Russell MJ. 2015. Mackinawite and greigite in ancient alkaline hydrothermal chimneys: identifying potential key catalysts for emergent life. Earth Planet Sci Lett 430:105–114. doi:10.1016/j.epsl.2015.08.013

[7] Hermis N, LeBlanc G, Barge LM. 2021. Simulation of early Earth hydrothermal chimneys in a thermal gradient environment. J Vis Exp. doi:10.3791/61789 33720128

[8] Carman NA, Hausrath EM, Celestian A, Chavez J, Hermis N, LaRowe DE, Fraeman AA, Sheppard RY, Adcock CT, Tschauner O, Rampe EB, Price R, Barge LM. 2024. Fe/Mg-silicate chemical gardens as analogs to silicate-rich hydrothermal chimneys on early Earth and Mars. ACS Earth Space Chem 8:1982–1996. doi:10.1021/acsearthspacechem.4c00109

[9] Dalton JB, Mogul R, Kagawa HK, Chan SL, Jamieson CS. 2003. Near-infrared detection of potential evidence for microscopic organisms on Europa. Astrobiology 3:505–529. doi:10.1089/153110703322610618 14678661

[10] Dickson J, Martinez E, Pagano JJ, Hudson R, Perl SM, Barge LM. 2022. Incorporating microbes into laboratory-grown chimneys for hydrothermal microbiology experiments. ACS Earth Space Chem 6:953–961. doi:10.1021/acsearthspacechem.1c00354

[11] Harris RL, Schuerger AC. 2025. Hydrogenotrophic methanogenesis at 7-12 mbar by *Methanosarcina barkeri* under simulated martian atmospheric conditions. Sci Rep 15:2880. doi:10.1038/s41598-025-86145-1 PMC11754898 39843490

[12] Hopton CM, Nienow P, Cockell CS. 2025. Growth, physiology, and metabolism of *Halomonas meridiana* in aqueous ammonium sulfate with implications for icy moon astrobiology. Front Microbiol 16:1642998. doi:10.3389/fmicb.2025.1642998 PMC12492958 41048487

[13] Tran PQ, Anantharaman K. 2021. Biogeochemistry goes viral: towards a multifaceted approach to study viruses and biogeochemical cycling. mSystems 6:e01138–21. doi:10.1128/msystems.01138-21 PMC8510517 34636672

[14] Danovaro R, Dell’Anno A, Corinaldesi C, Rastelli E, Cavicchioli R, Krupovic M, Noble RT, Nunoura T, Prangishvili D. 2016. Virus-mediated archaeal hecatomb in the deep seafloor. Sci Adv 2:e1600492. doi:10.1126/sciadv.1600492 PMC5061471 27757416

[15] Mastascusa V, Romano I, Di Donato P, Poli A, Della Corte V, Rotundi A, Bussoletti E, Quarto M, Pugliese M, Nicolaus B. 2014. Extremophiles survival to simulated space conditions: an astrobiology model study. Orig Life Evol Biosph 44:231–237. doi:10.1007/s11084-014-9397-y PMC4669584 25573749

[16] Vance SD, Craft KL, Shock E, Schmidt BE, Lunine J, Hand KP, McKinnon WB, Spiers EM, Chivers C, Lawrence JD, et al.. 2023. Investigating Europa’s Habitability with the Europa clipper. Space Sci Rev 219:81. doi:10.1007/s11214-023-01025-2 PMC10687213 38046182

[17] Saggio D, Phung CL, Bravo M, Corcoran TC, Snyder JC, Stieber SCE. 2026. Probing the habitability of potential sulfuric acid rich subsurface lakes in Europa’s ice shell via *Saci*/STIV integrated models. Front Microbiol 17:1849852. doi:10.3389/fmicb.2026.1849852 PMC13260153 42293565

[18] Rice G, Tang L, Stedman K, Roberto F, Spuhler J, Gillitzer E, Johnson JE, Douglas T, Young M. 2004. The structure of a thermophilic archaeal virus shows a double-stranded DNA viral capsid type that spans all domains of life. Proc Natl Acad Sci USA 101:7716–7720. doi:10.1073/pnas.0401773101 PMC419672 15123802

[19] Krupovic M, Prangishvili D, Hendrix RW, Bamford DH. 2011. Genomics of bacterial and archaeal viruses: dynamics within the prokaryotic virosphere. Microbiol Mol Biol Rev 75:610–635. doi:10.1128/MMBR.00011-11 PMC3232739 22126996

[20] Overton MS, Manuel RD, Lawrence CM, Snyder JC. 2023. Viruses of the *Turriviridae*: an emerging model system for studying archaeal virus-host interactions. Front Microbiol 14:1258997. doi:10.3389/fmicb.2023.1258997 PMC10551542 37808280

[21] Manuel RD, Snyder JC. 2024. The expanding diversity of viruses from extreme environments. Int J Mol Sci 25:3137. doi:10.3390/ijms25063137 PMC10970483 38542111

[22] Carr MH, Belton MJS, Chapman CR, Davies ME, Geissler P, Greenberg R, McEwen AS, Tufts BR, Greeley R, Sullivan R, Head JW, Pappalardo RT, Klaasen KP, Johnson TV, Kaufman J, Senske D, Moore J, Neukum G, Schubert G, Burns JA, Thomas P, Veverka J. 1998. Evidence for a subsurface ocean on Europa. Nature 391:363–365. doi:10.1038/34857 9450749

[23] Pappalardo RT, Buratti BJ, Korth H, Senske DA, Blaney DL, Blankenship DD, Burch JL, Christensen PR, Kempf S, Kivelson MG, et al.. 2024. Science overview of the Europa clipper mission. Space Sci Rev 220:40. doi:10.1007/s11214-024-01070-5

[24] Hendrix AR, Hurford TA, Barge LM, Bland MT, Bowman JS, Brinckerhoff W, Buratti BJ, Cable ML, Castillo-Rogez J, Collins GC, et al.. 2019. The NASA roadmap to ocean worlds. Astrobiology 19:1–27. doi:10.1089/ast.2018.1955 PMC6338575 30346215

[25] Waite JH, Glein CR, Perryman RS, Teolis BD, Magee BA, Miller G, Grimes J, Perry ME, Miller KE, Bouquet A, Lunine JI, Brockwell T, Bolton SJ. 2017. Cassini finds molecular hydrogen in the Enceladus plume: evidence for hydrothermal processes. Science 356:155–159. doi:10.1126/science.aai8703 28408597

[26] Postberg F, Khawaja N, Abel B, Choblet G, Glein CR, Gudipati MS, Henderson BL, Hsu H-W, Kempf S, Klenner F, Moragas-Klostermeyer G, Magee B, Nölle L, Perry M, Reviol R, Schmidt J, Srama R, Stolz F, Tobie G, Trieloff M, Waite JH. 2018. Macromolecular organic compounds from the depths of Enceladus. Nature 558:564–568. doi:10.1038/s41586-018-0246-4 PMC6027964 29950623

[27] Waite Jr JH, Lewis WS, Magee BA, Lunine JI, McKinnon WB, Glein CR, Mousis O, Young DT, Brockwell T, Westlake J, Nguyen M-J, Teolis BD, Niemann HB, McNutt Jr RL, Perry M, Ip W-H. 2009. Liquid water on Enceladus from observations of ammonia and 40Ar in the plume. Nature 460:487–490. doi:10.1038/nature08153

[28] Postberg F, Sekine Y, Klenner F, Glein CR, Zou Z, Abel B, Furuya K, Hillier JK, Khawaja N, Kempf S, Noelle L, Saito T, Schmidt J, Shibuya T, Srama R, Tan S. 2023. Detection of phosphates originating from Enceladus’s ocean. Nature 618:489–493. doi:10.1038/s41586-023-05987-9 PMC10266972 37316718

[29] Hsu H-W, Postberg F, Sekine Y, Shibuya T, Kempf S, Horányi M, Juhász A, Altobelli N, Suzuki K, Masaki Y, Kuwatani T, Tachibana S, Sirono S, Moragas-Klostermeyer G, Srama R. 2015. Ongoing hydrothermal activities within Enceladus. Nature 519:207–210. doi:10.1038/nature14262 25762281

[30] Russell MJ, Hall AJ, Martin W. 2010. Serpentinization as a source of energy at the origin of life. Geobiology 8:355–371. doi:10.1111/j.1472-4669.2010.00249.x 20572872

[31] Szalay JR, Allegrini F, Ebert RW, Bagenal F, Bolton SJ, Fatemi S, McComas DJ, Pontoni A, Saur J, Smith HT, Strobel DF, Vance SD, Vorburger A, Wilson RJ. 2024. Oxygen production from dissociation of Europa’s water-ice surface. Nat Astron 8:567–576. doi:10.1038/s41550-024-02206-x PMC11111413 38798715

[32] Spiers EM, Schmidt BE. 2023. Variable salinity and hydrogen production in Europa’s Ocean. JGR Planets 128:e2023JE008028. doi:10.1029/2023JE008028

[33] Bézard B, Vinatier S. 2020. On the H_2_ abundance and ortho-to-para ratio in Titan’s troposphere. Icarus 344:113261. doi:10.1016/j.icarus.2019.03.038

[34] Strobel DF. 2010. Molecular hydrogen in Titan’s atmosphere: implications of the measured tropospheric and thermospheric mole fractions. Icarus 208:878–886. doi:10.1016/j.icarus.2010.03.003

[35] Pappalardo RT, Head JW, Greeley R, Sullivan RJ, Pilcher C, Schubert G, Moore WB, Carr MH, Moore JM, Belton MJ, Goldsby DL. 1998. Geological evidence for solid-state convection in Europa’s ice shell. Nature 391:365–368. doi:10.1038/34862 9450750

[36] Hand KP, Carlson RW, Chyba CF. 2007. Energy, chemical disequilibrium, and geological constraints on Europa. Astrobiology 7:1006–1022. doi:10.1089/ast.2007.0156 18163875

[37] Pasek MA, Greenberg R. 2012. Acidification of Europa’s subsurface ocean as a consequence of oxidant delivery. Astrobiology 12:151–159. doi:10.1089/ast.2011.0666 22283235

[38] Carlson RW, Johnson RE, Anderson MS. 1999. Sulfuric acid on Europa and the radiolytic sulfur cycle. Science 286:97–99. doi:10.1126/science.286.5437.97 10506568

[39] Baidya AS, Gehringer MM, Savaniu C, Heubeck C, Stüeken EE. 2025. Magmatic and thermally produced reactive phosphorus 3.2 billion years ago and its implications for early life. Commun Earth Environ 6:895. doi:10.1038/s43247-025-02824-x PMC12615253 41245528

[40] Mojzsis SJ, Arrhenius G, McKeegan KD, Harrison TM, Nutman AP, Friend CR, New Collective Author. 1996. Evidence for life on Earth before 3,800 million years ago. Nature 384:55–59. doi:10.1038/384055a0 8900275

[41] Mastrogiuseppe M, Poggiali V, Hayes AG, Lunine JI, Seu R, Mitri G, Lorenz RD. 2019. Deep and methane-rich lakes on Titan. Nat Astron 3:535–542. doi:10.1038/s41550-019-0714-2

[42] Waite JH, Young DT, Cravens TE, Coates AJ, Crary FJ, Magee B, Westlake J. 2007. The process of tholin formation in Titan’s upper atmosphere. Science 316:870–875. doi:10.1126/science.1139727 17495166

[43] Gautier T. 2024. Reanalysis of the huygens GCMS dataset - I. High-resolution methane vertical profile in the atmosphere of Titan. Astron Astrophys 690:A165. doi:10.1051/0004-6361/202244583

[44] Taubner R-S, Pappenreiter P, Zwicker J, Smrzka D, Pruckner C, Kolar P, Bernacchi S, Seifert AH, Krajete A, Bach W, Peckmann J, Paulik C, Firneis MG, Schleper C, Rittmann SK-MR. 2018. Biological methane production under putative Enceladus-like conditions. Nat Commun 9:748. doi:10.1038/s41467-018-02876-y PMC5829080 29487311

[45] Singleton CM, McCalley CK, Woodcroft BJ, Boyd JA, Evans PN, Hodgkins SB, Chanton JP, Frolking S, Crill PM, Saleska SR, Rich VI, Tyson GW. 2018. Methanotrophy across a natural permafrost thaw environment. ISME J 12:2544–2558. doi:10.1038/s41396-018-0065-5 PMC6155033 29955139

[46] Farnsworth KK, McLain HL, Chung A, Trainer MG. 2024. Understanding Titan’s prebiotic chemistry: synthesizing amino acids through aminonitrile alkaline hydrolysis. ACS Earth Space Chem 8:2380–2392. doi:10.1021/acsearthspacechem.4c00114 PMC11664644 39720226

[47] Affholder A, Higgins PM, Cockell CS, Neish C, Soderlund KM, Malaska MJ, Farnsworth KK, Lopes RMC, Nixon CA, Melwani Daswani M, Miller KE, Sotin C. 2025. The viability of glycine fermentation in Titan’s subsurface Ocean. Planet Sci J 6:86. doi:10.3847/PSJ/adbc66

[48] Niemann HB, Atreya SK, Bauer SJ, Carignan GR, Demick JE, Frost RL, Gautier D, Haberman JA, Harpold DN, Hunten DM, Israel G, Lunine JI, Kasprzak WT, Owen TC, Paulkovich M, Raulin F, Raaen E, Way SH. 2005. The abundances of constituents of Titan’s atmosphere from the GCMS instrument on the Huygens probe. Nature 438:779–784. doi:10.1038/nature04122 16319830

[49] Brown ME, Hand KP. 2013. Salts and radiation products on the surface of Europa. AJ 145:110. doi:10.1088/0004-6256/145/4/110

[50] Hand KP, Brown ME. 2013. Keck II observations of hemispherical differences in H2O2 ON Europa. Astrophys J Lett 766:L21. doi:10.1088/2041-8205/766/2/L21

[51] Byrne PK, Dawson HG, Klimczak C, Regensburger PV, Crane KT, Catalano JG, Elder CM, Foley BJ, German CR, Green AP, Hemingway DJ, Melwani Daswani M, Panning MP, Randolph-Flagg N, Sherwood Lollar B, Skemer P, Vance SD, Wiens DA. 2026 Little to no active faulting likely at Europa’s seafloor today. Nat Commun 17. doi:10.1038/s41467-025-67151-3 PMC12775069 41495054

[52] Nelson RM, Kamp LW, Matson DL, Irwin PGJ, Baines KH, Boryta MD, Leader FE, Jaumann R, Smythe WD, Sotin C, et al.. 2009. Saturn’s Titan: surface change, ammonia, and implications for atmospheric and tectonic activity. Icarus 199:429–441. doi:10.1016/j.icarus.2008.08.013

[53] Chivers CJ, Buffo JJ, Schmidt BE. 2021. Thermal and chemical evolution of small, shallow water bodies in Europa’s ice shell. JGR Planets 126:e2020JE006692. doi:10.1029/2020JE006692

[54] Sahai N, LaRowe D, Senko JM. 2024. Bioenergetics of iron snow fueling life on Europa. Proc Natl Acad Sci USA 121:e2316452121. doi:10.1073/pnas.2316452121 PMC11047109 38621125

[55] Petricca F, Vance SD, Parisi M, Buccino D, Cascioli G, Castillo-Rogez J, Downey BG, Nimmo F, Tobie G, Journaux B, Magnanini A, Jones U, Panning M, Bagheri A, Genova A, Lunine JI. 2025. Titan’s strong tidal dissipation precludes a subsurface ocean. Nature 648:556–561. doi:10.1038/s41586-025-09818-x PMC12711566 41407902

[56] Sotin C, Head JW III, Tobie G. 2002. Europa: tidal heating of upwelling thermal plumes and the origin of lenticulae and chaos melting. Geophys Res Lett 29:74–1 doi:10.1029/2001GL013844

[57] Russell MJ, Murray AE, Hand KP. 2017. The possible emergence of life and differentiation of a shallow biosphere on irradiated icy worlds: the example of Europa. Astrobiology 17:1265–1273. doi:10.1089/ast.2016.1600 PMC5729856 29016193

[58] Anderson JD, Schubert G, Jacobson RA, Lau EL, Moore WB, Sjogren WL. 1998. Europa’s differentiated internal structure: inferences from four Galileo encounters. Science 281:2019–2022. doi:10.1126/science.281.5385.2019 9748159

[59] Zolotov MY, Shock EL. 2004. A model for low‐temperature biogeochemistry of sulfur, carbon, and iron on Europa. J Geophys Res 109. doi:10.1029/2003JE002194

[60] Weber JM, Marlin TC, Prakash M, Teece BL, Dzurilla K, Barge LM. 2023. A review on hypothesized metabolic pathways on Europa and Enceladus: space-flight detection considerations. Life (Basel) 13:1726. doi:10.3390/life13081726 PMC10456045 37629583

[61] Kang W, Mittal T, Bire S, Campin J-M, Marshall J. 2022. How does salinity shape ocean circulation and ice geometry on Enceladus and other icy satellites? Sci Adv 8:eabm4665. doi:10.1126/sciadv.abm4665 PMC9299536 35857831

[62] Kivelson MG, Khurana KK, Russell CT, Volwerk M, Walker RJ, Zimmer C. 2000. Galileo magnetometer measurements: a stronger case for a subsurface ocean at Europa. Science 289:1340–1343. doi:10.1126/science.289.5483.1340 10958778

[63] Leitner MA, Lunine JI. 2019. Modeling early Titan’s ocean composition. Icarus 333:61–70. doi:10.1016/j.icarus.2019.05.008

[64] Lopes RMC, Kirk RL, Mitchell KL, LeGall A, Barnes JW, Hayes A, Kargel J, Wye L, Radebaugh J, Stofan ER, Janssen MA, Neish CD, Wall SD, Wood CA, Lunine JI, Malaska MJ. 2013. Cryovolcanism on titan: new results from cassini RADAR and VIMS. JGR Planets 118:416–435. doi:10.1002/jgre.20062

[65] Ray C, Glein CR, Waite JH, Teolis B, Hoehler T, Huber JA, Lunine J, Postberg F. 2021. Oxidation processes diversify the metabolic menu on Enceladus. Icarus 364:114248. doi:10.1016/j.icarus.2020.114248

[66] MacKenzie SM. 2020. Encealdus orbilander: a flagship mission concept for astrobiology

[67] Johnson RE, Nelson ML, McCord TB, Gradie JC. 1988. Analysis of Voyager images of Europa: plasma bombardment. Icarus 75:423–436. doi:10.1016/0019-1035(88)90155-8

[68] Lemasquerier DG, Bierson CJ, Soderlund KM. 2023. Europa’s Ocean translates interior tidal heating patterns to the ice‐ocean boundary. AGU Advances 4:e2023AV000994. doi:10.1029/2023AV000994

[69] McCollom TM. 1999. Methanogenesis as a potential source of chemical energy for primary biomass production by autotrophic organisms in hydrothermal systems on Europa. J Geophys Res 104:30729–30742. doi:10.1029/1999JE001126

[70] Brock TD, Brock KM, Belly RT, Weiss RL. 1972*.* *Sulfolobus*: a new genus of sulfur-oxidizing bacteria living at low pH and high temperature. Archiv Mikrobiol 84:54–68. doi:10.1007/BF00408082 4559703

[71] Russell MJ, Barge LM, Bhartia R, Bocanegra D, Bracher PJ, Branscomb E, Kidd R, McGlynn S, Meier DH, Nitschke W, Shibuya T, Vance S, White L, Kanik I. 2014. The drive to life on wet and icy worlds. Astrobiology 14:308–343. doi:10.1089/ast.2013.1110 PMC3995032 24697642

[72] He C, Serigano J, Hörst SM, Radke M, Sebree JA. 2022. Titan atmospheric chemistry revealed by low-temperature N_2_–CH_4_ plasma discharge experiments. ACS Earth Space Chem 6:2295–2304. doi:10.1021/acsearthspacechem.2c00164

[73] Saggio D, Phung CL, Bravo M, Corcoran TC, Snyder JC, Stieber SCE. 2026. Probing the habitability of sulfuric acid rich subsurface lakes in Europa’s ice shell via *Sulfolobus*/STIV integrated biogeochemical models. ChemRxiv. doi:10.26434/chemrxiv.15001096/v1 PMC13260153 42293565

[74] Iwasaki T. 2010. Iron-sulfur world in aerobic and hyperthermoacidophilic archaea *Sulfolobus*. Archaea 2010:1–14. doi:10.1155/2010/842639 PMC2946596 20885930

[75] Brock TD, Gustafson J. 1976. Ferric iron reduction by sulfur- and iron-oxidizing bacteria. Appl Environ Microbiol 32:567–571. doi:10.1128/aem.32.4.567-571.1976 PMC170307 825043

[76] Russell MJ, Hall AJ, Cairns-smith AG, Braterman PS. 1988. Submarine hot springs and the origin of life. Nature 336:117–117. doi:10.1038/336117a0

[77] Hatton B, Rickard D. 2008. Nucleic acids bind to nanoparticulate iron (II) monosulphide in aqueous solutions. Orig Life Evol Biosph 38:257–270. doi:10.1007/s11084-008-9132-7 18409029

[78] Sakai HD, Kurosawa N. 2018. *Saccharolobus caldissimus* gen. nov., sp. nov., a facultatively anaerobic iron-reducing hyperthermophilic archaeon isolated from an acidic terrestrial hot spring, and reclassification of *Sulfolobus solfataricus* as *Saccharolobus solfataricus* comb. nov. and *Sulfolobus shibatae* as *Saccharolobus shibatae* comb. nov. Int J Syst Evol Microbiol 68:1271–1278. doi:10.1099/ijsem.0.002665 29485400

[79] Barge LM, Abedian Y, Doloboff IJ, Nuñez JE, Russell MJ, Kidd RD, Kanik I. 2015. Chemical gardens as flow-through reactors simulating natural hydrothermal systems. J Vis Exp e53015:53015. doi:10.3791/53015 PMC4692733 26650915

[80] Trinh KT, Bierson CJ, O’Rourke JG. 2023. Slow evolution of Europa’s interior: metamorphic ocean origin, delayed metallic core formation, and limited seafloor volcanism. Sci Adv 9:eadf3955. doi:10.1126/sciadv.adf3955 PMC10275592 37327336

[81] Moreras-Marti A, Fox-Powell M. 2022. Sulfur isotopes as biosignatures for Mars and Europa exploration. Journal of Geological Society, 179. doi:10.1144/jgs2021-134

[82] White LM, Shibuya T, Vance SD, Christensen LE, Bhartia R, Kidd R, Hoffmann A, Stucky GD, Kanik I, Russell MJ. 2020. Simulating serpentinization as it could apply to the emergence of life using the JPL hydrothermal reactor. Astrobiology 20:307–326. doi:10.1089/ast.2018.1949 32125196

[83] Baker BJ, De Anda V, Seitz KW, Dombrowski N, Santoro AE, Lloyd KG. 2020. Diversity, ecology and evolution of Archaea. Nat Microbiol 5:887–900. doi:10.1038/s41564-020-0715-z 32367054

[84] Cozen AE, Weirauch MT, Pollard KS, Bernick DL, Stuart JM, Lowe TM. 2009. Transcriptional map of respiratory versatility in the hyperthermophilic crenarchaeon *Pyrobaculum aerophilum*. J Bacteriol 191:782–794. doi:10.1128/JB.00965-08 PMC2632070 19047344

[85] Winkel M, Mitzscherling J, Overduin PP, Horn F, Winterfeld M, Rijkers R, Grigoriev MN, Knoblauch C, Mangelsdorf K, Wagner D, Liebner S. 2018. Anaerobic methanotrophic communities thrive in deep submarine permafrost. Sci Rep 8:1291. doi:10.1038/s41598-018-19505-9 PMC5778128 29358665

[86] Fernandes-Martins MC, Colman DR, Boyd ES. 2024. Sulfide oxidation by members of the *Sulfolobales*. PNAS Nexus 3:gae201. doi:10.1093/pnasnexus/pgae201 PMC11143483 38827816

[87] Baquero DP, Contursi P, Piochi M, Bartolucci S, Liu Y, Cvirkaite-Krupovic V, Prangishvili D, Krupovic M. 2020. New virus isolates from Italian hydrothermal environments underscore the biogeographic pattern in archaeal virus communities. ISME J 14:1821–1833. doi:10.1038/s41396-020-0653-z PMC7305311 32322010

[88] Liu L-J, Jiang Z, Wang P, Qin Y-L, Xu W, Wang Y, Liu S-J, Jiang C-Y. 2021. Physiology, taxonomy, and sulfur metabolism of the sulfolobales, an order of thermoacidophilic archaea. Front Microbiol 12:768283. doi:10.3389/fmicb.2021.768283 PMC8551704 34721370

[89] Bischof LF, Haurat MF, Hoffmann L, Albersmeier A, Wolf J, Neu A, Pham TK, Albaum SP, Jakobi T, Schouten S, Neumann-Schaal M, Wright PC, Kalinowski J, Siebers B, Albers S-V. 2019. Early response of sulfolobus acidocaldarius to nutrient limitation. Front Microbiol 9:3201. doi:10.3389/fmicb.2018.03201 PMC6335949 30687244

[90] Fernandes‐Martins MC, Springer C, Colman DR, Boyd ES. 2024. Acquisition of elemental sulfur by sulfur‐oxidising *Sulfolobales*. Environ Microbiol 26:e16691. doi:10.1111/1462-2920.16691 39206712

[91] Davila AF, Eigenbrode JL. 2024. Enceladus: astrobiology revisited. JGR Biogeo Sci 129. doi:10.1029/2023JG007677

[92] Feinberg LF, Srikanth R, Vachet RW, Holden JF. 2008. Constraints on anaerobic respiration in the hyperthermophilic archaea *Pyrobaculum islandicum* and *Pyrobaculum aerophilum*. Appl Environ Microbiol 74:396–402. doi:10.1128/AEM.02033-07 PMC2223247 18039820

[93] Kashefi K, Lovley DR. 2000. Mn(IV), and toxic metals at 100°C by *Pyrobaculum islandicum*. Appl Environ Microbiol 66:1050–1056. doi:10.1128/AEM.66.3.1050-1056.2000 PMC91941 10698770

[94] Koblitz J. 2024. 611: Pyrobaculum (BS) Medium. MediaDive. Available from: https://mediadive.dsmz.de

[95] Koblitz J. 2024. 390: pyrobaculum medium. MediaDive. Available from: https://mediadive.dsmz.de

[96] Gomez-Buckley AC, Showalter GM, Wong ML. 2022. Modeling virus and bacteria populations in Europa’s subsurface Ocean. Life (Basel) 12:620. doi:10.3390/life12050620 PMC9147769 35629289

[97] Quehenberger J, Shen L, Albers S-V, Siebers B, Spadiut O. 2017. Sulfolobus – a potential key organism in future biotechnology. Front Microbiol 8:2474. doi:10.3389/fmicb.2017.02474 PMC5733018 29312184

[98] Order: Sulfolobus acidocaldarius: 33909. 2025. ATCC. Available from: https://www.atcc.org/products/33909

[99] Koblitz J. 2024. 88a: Sulfolobus Medium (Anaerobic). MediaDive. Available from: https://mediadive.dsmz.de

[100] Ramirez-Llodra et. al., E. 2023. Hot vents beneath an icy ocean: the aurora vent field, gakkel ridge. Oceanography, 36 (1):6–17. doi:10.5670/oceanog.2023.103

[101] Lin TJ, Ver Eecke HC, Breves EA, Dyar MD, Jamieson JW, Hannington MD, Dahle H, Bishop JL, Lane MD, Butterfield DA, Kelley DS, Lilley MD, Baross JA, Holden JF. 2016. Linkages between mineralogy, fluid chemistry, and microbial communities within hydrothermal chimneys from the Endeavour Segment, Juan de Fuca Ridge. Geochem Geophys Geosyst 17:300–323. doi:10.1002/2015GC006091 PMC6094386 30123099

[102] Hesse MA, Jordan JS, Vance SD, Oza AV. 2022. Downward oxidant transport through Europa’s ice shell by density‐driven brine percolation. Geophys Res Lett 49:e2021GL095416. doi:10.1029/2021GL095416

[103] Ye X, van der Does C, Albers S-V. 2020. SaUspA, the universal stress protein of sulfolobus acidocaldarius stimulates the activity of the PP2A phosphatase and is involved in growth at high salinity. Front Microbiol 11:12:898185. doi:10.3389/fmicb.2020.598821 PMC7693658 33304342

[104] Simon G, Walther J, Zabeti N, Combet-Blanc Y, Auria R, van der Oost J, Casalot L. 2009. Effect of O_2_ concentrations on *Sulfolobus solfataricus* P2. FEMS Microbiol Lett 299:255–260. doi:10.1111/j.1574-6968.2009.01759.x 19735462

[105] Hjort K, Bernander R. 1999. Changes in cell size and DNA content in *Sulfolobus* cultures during dilution and temperature shift experiments. J Bacteriol 181:5669–5675. doi:10.1128/JB.181.18.5669-5675.1999 PMC94086 10482507

[106] Decho AW, Gutierrez T. 2017. Microbial extracellular polymeric substances (EPSs) in Ocean systems. Front Microbiol 8:922. doi:10.3389/fmicb.2017.00922 PMC5445292 28603518

[107] Marx JG, Carpenter SD, Deming JW. 2009. Production of cryoprotectant extracellular polysaccharide substances (EPS) by the marine psychrophilic bacterium *Colwellia psychrerythraea* strain 34H under extreme conditions. This article is one of a selection of papers in the Special Issue on Polar and Alpine Microbiology. Can J Microbiol 55:63–72. doi:10.1139/W08-130 19190702

[108] Carrión O, Delgado L, Mercade E. 2015. New emulsifying and cryoprotective exopolysaccharide from Antarctic *Pseudomonas* sp. ID1. Carbohydr Polym 117:1028–1034. doi:10.1016/j.carbpol.2014.08.060 25498731

[109] Yin W, Wang Y, Liu L, He J. 2019. Biofilms: the microbial “Protective clothing” in extreme environments. IJMS 20:3423. doi:10.3390/ijms20143423 PMC6679078 31336824

[110] Park HR, Lee JH, Ji HJ, Lim S, Ahn KB, Seo HS. 2022. Radioprotection of deinococcal exopolysaccharide BRD125 by regenerating hematopoietic stem cells. Front Oncol 12:e898185. doi:10.3389/fonc.2022.898185 PMC9549790 36226052

[111] Niemira BA. 2007. Irradiation sensitivity of planktonic and biofilm-associated *Escherichia coli* O157:H7 isolates is influenced by culture conditions. Appl Environ Microbiol 73:3239–3244. doi:10.1128/AEM.02764-06 PMC1907117 17384305

[112] Ida T, Sawa T, Ihara H, Tsuchiya Y, Watanabe Y, Kumagai Y, Suematsu M, Motohashi H, Fujii S, Matsunaga T, Yamamoto M, Ono K, Devarie-Baez NO, Xian M, Fukuto JM, Akaike T. 2014. Reactive cysteine persulfides and S-polythiolation regulate oxidative stress and redox signaling. Proc Natl Acad Sci USA 111:7606–7611. doi:10.1073/pnas.1321232111 PMC4040604 24733942

[113] Bills BG, Nimmo F. 2011. Rotational dynamics and internal structure of Titan. Icarus 214:351–355. doi:10.1016/j.icarus.2011.04.028

[114] Baland R-M, Van Hoolst T, Yseboodt M, Karatekin Ö. 2011. Titan’s obliquity as evidence of a subsurface Ocean? A&A 530:A141. doi:10.1051/0004-6361/201116578

[115] Biwer P, Neumann-Schaal M, Henke P, Jahn D, Schulz S. 2022. Thiol metabolism and volatile metabolome of *Clostridioides difficile*. Front Microbiol 13:864587. doi:10.3389/fmicb.2022.864587 PMC9243749 35783419

[116] Stoker CR, Boston PJ, Mancinelli RL, Segal W, Khare BN, Sagan C, New Collective Author. 1990. Microbial metabolism of tholin. Icarus 85:241–256. doi:10.1016/0019-1035(90)90114-o 11538367

[117] Slobodkina GB, Kolganova TV, Tourova TP, Kostrikina NA, Jeanthon C, Bonch-Osmolovskaya EA, Slobodkin AI. 2008. *Clostridium tepidiprofundi* sp. nov., a moderately thermophilic bacterium from a deep-sea hydrothermal vent. Int J Syst Evol Microbiol 58:852–855. doi:10.1099/ijs.0.65485-0 18398181

[118] Kelly LC, Cockell CS, Summers S. 2012. Diverse microbial species survive high ammonia concentrations. Int J Astrobiology 11:125–131. doi:10.1017/S147355041200002X

[119] Raatjes GJM, Smelt J. 1979. *Clostridium botulinum* can grow and form toxin at pH values lower than 4.6. Nature 281:398–399. doi:10.1038/281398a0 39257

[120] Wetzel D, McBride SM. 2020. The Impact of pH on *Clostridioides difficile* sporulation and physiology. Appl Environ Microbiol 86:e02706-19. doi:10.1128/AEM.02706-19 PMC6997743 31811041

[121] de Jong AEI, Rombouts FM, Beumer RR. 2004. Behavior of *Clostridium perfringens* at low temperatures. Int J Food Microbiol 97:71–80. doi:10.1016/j.ijfoodmicro.2004.03.030 15527920

[122] Fernández-Naveira Á, Veiga MC, Kennes C. 2019. Effect of salinity on C1-gas fermentation by *Clostridium carboxidivorans* producing acids and alcohols. AMB Express 9:110. doi:10.1186/s13568-019-0837-y PMC6637162 31317357

[123] Miller KE, Glein CR, Waite JH. 2019. Contributions from accreted organics to Titan’s atmosphere: new insights from cometary and chondritic data. ApJ 871:59. doi:10.3847/1538-4357/aaf561

[124] Nixon CA. 2024. The composition and chemistry of Titan’s atmosphere. ACS Earth Space Chem 8:406–456. doi:10.1021/acsearthspacechem.2c00041 PMC10961852 38533193

[125] Bock AK, Schönheit P. 1995. Growth of *Methanosarcina barkeri* (Fusaro) under nonmethanogenic conditions by the fermentation of pyruvate to acetate: ATP synthesis via the mechanism of substrate level phosphorylation. J Bacteriol 177:2002–2007. doi:10.1128/jb.177.8.2002-2007.1995 PMC176842 7721692

[126] Ngoumelah D, Harnisch F, Sulheim S, Heggeset TMB, Aune IH, Wentzel A, Kretzschmar J. 2022. A unified and simple medium for growing model methanogens. Front Microbiol 13:e1046260. doi: 10.3389/fmicb.2022.1046260 10.3389/fmicb.2022.1046260 PMC9871610 36704566

[127] Dai X, Yan H, Li N, He J, Ding Y, Dai L, Dong B. 2016. Metabolic adaptation of microbial communities to ammonium stress in a high solid anaerobic digester with dewatered sludge. Sci Rep 6:28193. doi:10.1038/srep28193 PMC4911566 27312792

[128] Wu W, Zheng L, Holmstrand H, Tarbier B, Wild B, Shakhova N, Kosmach D, Semiletov I, Brüchert V, Xu Y, Gustafsson Ö. 2026. Intensive methane seep region on the outer East Siberian Arctic Shelf shows biomarker evidence for aerobic oxidation of methane. Org Geochem 215:105171. doi:10.1016/j.orggeochem.2026.105171

[129] Schütte UME, Cadieux SB, Hemmerich C, Pratt LM, White JR. 2016. Unanticipated geochemical and microbial community structure under seasonal ice cover in a dilute, dimictic Arctic lake. Front Microbiol 7:1035. doi:10.3389/fmicb.2016.01035 PMC4932660 27458438

[130] Miyajima Y, Aoyagi T, Yoshioka H, Hori T, Takahashi HA, Tanaka M, Tsukasaki A, Goto S, Suzumura M. 2024. Impact of concurrent aerobic–anaerobic methanotrophy on methane emission from marine sediments in gas hydrate area. Environ Sci Technol 58:4979–4988. doi:10.1021/acs.est.3c09484 PMC10956523 38445630

[131] Schreiber L, Holler T, Knittel K, Meyerdierks A, Amann R. 2010. Identification of the dominant sulfate‐reducing bacterial partner of anaerobic methanotrophs of the ANME‐2 clade. Environ Microbiol 12:2327–2340. doi:10.1111/j.1462-2920.2010.02275.x 21966923

[132] Koblitz J. 2024. 120a: Methanosarcina Barkeri Medium. MediaDive. Available from: https://mediadive.dsmz.de

[133] Hippe H, Caspari D, Fiebig K, Gottschalk G. 1979. Utilization of trimethylamine and other N-methyl compounds for growth and methane formation by *Methanosarcina barkeri*. Proc Natl Acad Sci USA 76:494–498. doi:10.1073/pnas.76.1.494 PMC382968 284366

[134] Grubisic A, Trainer MG, Li X, Brinckerhoff WB, van Amerom FH, Danell RM, Costa JT, Castillo M, Kaplan D, Zacny K. 2021. Laser desorption mass spectrometry at Saturn’s moon Titan. Int J Mass Spectrom 470:116707. doi:10.1016/j.ijms.2021.116707

[135] Rensen EI, Mochizuki T, Quemin E, Schouten S, Krupovic M, Prangishvili D. 2016. A virus of hyperthermophilic archaea with a unique architecture among DNA viruses. Proc Natl Acad Sci USA 113:2478–2483. doi:10.1073/pnas.1518929113 PMC4780613 26884161

[136] Wang F, Baquero DP, Su Z, Beltran LC, Prangishvili D, Krupovic M, Egelman EH. 2020. The structures of two archaeal type IV pili illuminate evolutionary relationships. Nat Commun 11:3424. doi:10.1038/s41467-020-17268-4 PMC7347861 32647180

[137] Puddu M, Stark WJ, Grass RN. 2015. Silica microcapsules for long-term, robust, and reliable room temperature RNA preservation. Adv Healthc Mater 4:1332–1338. doi:10.1002/adhm.201500132 25899883

[138] Fanarraga ML, García Hevia L. 2026. Silica nanoparticles as advanced platforms for nucleic acid delivery. Materials Today Bio 37:102921. doi:10.1016/j.mtbio.2026.102921 PMC12925296 41732389

[139] Paunescu D, Puddu M, Soellner JOB, Stoessel PR, Grass RN. 2013. Reversible DNA encapsulation in silica to produce ROS-resistant and heat-resistant synthetic DNA “fossils”. Nat Protoc 8:2440–2448. doi:10.1038/nprot.2013.154 24202557

[140] Zeng Z, Liu X-L, Farley KR, Wei JH, Metcalf WW, Summons RE, Welander PV. 2019. GDGT cyclization proteins identify the dominant archaeal sources of tetraether lipids in the ocean. Proc Natl Acad Sci USA 116:22505–22511. doi:10.1073/pnas.1909306116 PMC6842593 31591189

[141] Roine E, Bamford DH. 2012. Lipids of archaeal viruses. Archaea 2012:384919. doi:10.1155/2012/384919 PMC3461281 23049284

[142] Brard M, Richter W, Benvegnu T, Plusquellec D. 2004. Synthesis and supramolecular assemblies of bipolar archaeal glycolipid analogues containing a cis-1,3-disubstituted cyclopentane ring. J Am Chem Soc 126:10003–10012. doi:10.1021/ja049805n 15303874

[143] Siliakus MF, van der Oost J, Kengen SWM. 2017. Adaptations of archaeal and bacterial membranes to variations in temperature, pH and pressure. Extremophiles 21:651–670. doi:10.1007/s00792-017-0939-x PMC5487899 28508135

[144] Anderson RE, Kouris A, Seward CH, Campbell KM, Whitaker RJ. 2017. Structured populations of sulfolobus acidocaldarius with susceptibility to mobile genetic elements. Genome Biol Evol 9:1699–1710. doi:10.1093/gbe/evx104 PMC5554439 28633403

[145] Wirth JF, Snyder JC, Hochstein RA, Ortmann AC, Willits DA, Douglas T, Young MJ. 2011. Development of a genetic system for the archaeal virus Sulfolobus turreted icosahedral virus (STIV). Virology 415:6–11. doi:10.1016/j.virol.2011.03.023 21496857

[146] Snyder JC, Young MJ. 2011. Potential role of cellular ESCRT proteins in the STIV life cycle. Biochem Soc Trans 39:107–110. doi:10.1042/BST0390107 21265756

[147] Howell SM, Pappalardo RT. 2020. NASA’s Europa clipper-a mission to a potentially habitable ocean world. Nat Commun 11:1311. doi:10.1038/s41467-020-15160-9 PMC7066167 32161262

[148] Weidenbach K, Nickel L, Neve H, Alkhnbashi OS, Künzel S, Kupczok A, Bauersachs T, Cassidy L, Tholey A, Backofen R, Schmitz RA. 2017. *Methanosarcina* spherical virus, a novel archaeal lytic virus targeting methanosarcina strains. J Virol 91:e00955-17. doi:10.1128/JVI.00955-17 PMC5660497 28878086

